# Book Reviews

**Published:** 1828-10

**Authors:** 


### CRITICAL ANALYSES.

Quæ laudanda forent, et quæ culpanda, vicissim

Illa, prius, cretâ; mox hæc, carbone, *notamus*.—Persius.

*A Compendium of the Diseases of the Human Eye; containing Descriptions and Explanations of the various Diseases, illustrated by Engravings, and accompanied by Practical Observations on their Treatment.* By Alexander Watson, Fellow of the Royal College of Surgeons, Edinburgh, &c. &c. &c. *Second Edition, much enlarged.*—8vo. pp. 192. Maclachlan and Stewart, Edinburgh, 1828.

This treatise is designed chiefly for the use of students. Its objects are to shew how the various diseases of the eye may be distinguished from each other, either by the appearances of the affected organ, or by these in connexion with other symptoms. Many of the diseases, though common, have neither been described nor delineated by any writer. The author trusts that the brief descriptions of diseases which he has given are accurate, as they are the result of his actual observation. He has been careful to notice every particular which he considered necessary, either towards forming a correct diagnosis, or in elucidating the nature of each disease. The treatment recommended has been found most successful after considerable experience. "Without attempting to expose or to combat prevailing errors, whether theoretical or practical, the author has stated what, either from experience, or after mature consideration and reflection, he conceives to be correct."

We are by no means inclined to commend an eager spirit of disputation, but we cannot entirely approve of the forbearance of an author who insinuates that he is conscious of the existence of "theoretical and practical" errors upon the subject to which he has directed his especial attention, but who yet allows those errors to pass unexamined and unrefuted. The grand object of all our labours must be to distinguish right from wrong, and we certainly ought not to hesitate in pointing out, for the general benefit, the inaccuracies which our reflection or experience has enabled us to detect in the doctrines of others. It is as incumbent upon a writer to perform this duty unreservedly, as to state with the most impartial candour the results of his own observation.

The treatment of diseases of the eye was formerly almost entirely confided to persons styling themselves *oculists*. These oculists not only laid no claim to a knowledge of any other branch of surgery, but they appeared to pride themselves upon their ignorance of those general pathological principles, without which they might certainly, by extensive practice, become expert operators; but the operative part of surgery is neither the most important to the practitioner, nor the most advantageous to the public. To perceive the first invasion of disease, and to cut it short by appropriate treatment, must be considered the most gratifying part of our duty; and, as this duty can only be performed by those who have studied the general laws of disease, it is very fortunate that, in the present day, ophthalmic surgery is almost entirely intrusted to those men who have been regularly educated in all branches of the profession, but who still have paid particular attention to diseases of the eye. " No one can expect to succeed as an oculist who is not well acquainted with the constitutional as well as local causes of disease, and with the effects of remedies as they operate on the general system."

*Ophthalmia, or Inflammation of the Eye.*—Inflammation is by far the most frequent disease that affects the eye; and most of the other affections of this organ are the consequences of inflammation, arising either from violence or improper treatment. The usual commencement and progress of the symptoms of " active or acute inflammation of the eye," which forms the subject of the first section, are too well known to require repetition. Swelling, Mr. Watson observes, only takes place when the eyelids, or parts surrounding the eyeball, are inflamed. In these cases, swelling sometimes takes place to a great degree. Redness is not a constant symptom. " The internal parts of the eye may be inflamed to a very violent degree without any external redness. The same may be said of intolerance of light, which, at the commencement of the disease, seldom occurs in proportion to the degree of inflammation, but depends rather on the nature of the parts affected." (P. 9.)

Pain, though a very common symptom of acute inflammation of the eye, is not always present.

" The pain may be very violent, darting through the head,—it may intermit,—it may amount only to increased heat and uneasiness in the organ,—or it may be wanting altogether, while the inflammation, attended with other symptoms, exists to a considerable degree. The coats of the eye are so unyielding in their nature, that when any of the more internal parts are inflamed the pain is very great." (P. 10.)

The most common local causes are—

" 1. Exposure to excessive heat and light. 2. Cold and moisture. 3. Wounds and other injuries of the eye. 4. The introduction of foreign bodies. 5. The application of acrid substances or acrid vapours to the eye. 6. Intemperance. 7. Contagion, by the application of morbid matter to the eye.

"The most common constitutional causes are—

"1. The suppression of customary evacuations. 2. General plethora. 3. Derangements of the alimentary canal.

"Acute inflammation of the eye occurs as a symptomatic affection in small-pox, measles, erysipelas, gonorrhœa, gout, rheumatism, scrofula, cancer, and syphilis. In many of these cases it requires to be treated as a local as well as a constitutional disease." (P. 12.)

*Treatment.*—From whatever cause it may have arisen, acute inflammation of the eye generally requires the same mode of treatment. Of course, when the inflammation has been caused by a foreign substance having got into the eye, that must first be entirely removed before any other means of cure are applied. If any fluid of an *acid* nature has entered the eye, or one which can be decomposed by lime-water, this may be used to wash the eye, if it can be immediately procured. If any substance has got within the eye, and can be seen, it should be abstracted by making an incision in the cornea, and introducing the forceps through it. In some cases this plan cannot be necessary, as the extraneous body may be removed by the wound inflicted upon its entrance into the eye. Bloodletting, both general and local, repeated according to the severity of the disease and the constitution of the patient, is the remedy chiefly to be relied on.

"In the case of an infant affected with ophthalmia, one leech may be applied to each eye; but the bleeding should be immediately stopped by compression, when the leech has separated: and this bleeding may be repeated in twenty-four or thirty-six hours after, according to circumstances." (P. 15.)

The local detraction of blood may be effected by making scarifications, or by applying two or three leeches upon the inner surface of the eyelids, when its vessels are enlarged, or by the application of leeches externally, or by cupping on the temples.

"Scarifications, or the application of leeches upon the inner surface of the eyelids, should not, in general, be employed, except in cases where the violence of the inflammation has been overcome by other treatment; for, when employed previously to this, they have generally been found to produce hurtful irritation. But they may be highly beneficial in cases where the inflammation has been in some measure subdued, and where some inflammation, with increased vascularity of the conjunctiva, still continues.

"The application of leeches to the neighbourhood of the eye is the most easy and gentle way of abstracting blood in slight cases of inflammation, where a small quantity may be quite sufficient. They form, likewise, a useful auxiliary to general bleeding in severe cases. The application of the leeches directly upon the external surface of the eyelids, is very liable to excite distressing erysipelatous inflammation of the parts, by which the employment of other remedies is prevented. It is preferable, therefore, to apply them either to the forehead, immediately above the eyebrow, or the cheek immediately below the lower eyelid. By applying them to the forehead, blood is taken directly from the branches of the ophthalmic artery; but the bites do not bleed so freely when the leeches are detached, as they do when they are applied under the lower eyelid, where the skin is more vascular." (P. 16.)

The antiphlogistic regimen, in the strictest sense of the term, is to be enforced. During the first or most acute stage of the disease, warm applications give most relief. They may be applied in the common manner, or in the form of vapour. The vapour employed may consist either of that arising from the decoction of poppies, hyoscyamus, or camomile flowers, or from boiling water poured upon a small quantity of camphor and tincture of opium. A pint of boiling water may be poured into a vessel containing one drachm of camphorated spirit of wine and one of tincture of opium. The vapour is applied merely by holding the eye open over the vessel in which the hot liquid is contained.

"When warm applications are employed, they should be discontinued whenever a remission of the inflammation takes place, and tepid or cold ones should then be substituted; for, when warm applications are continued longer than this, they are apt to cause too much relaxation of the blood-vessels, and thereby to induce passive inflammation." (P. 20.)

Mr. Watson's experience leads him to the same conclusions respecting the use of purgatives, emetics, diaphoretics, and blisters, which have been derived by other writers upon the same subject.

*Puncture of the cornea.*—"The evacuation of the aqueous humor by a puncture of the cornea was first proposed by Mr. Wardrop, from whose observations it appears that it may be performed with very great benefit in all cases of violent acute inflammation of the eyeball, and particularly when they resist other treatment. This operation is indicated when the pain is very intense, accompanied with a sense of fulness in the eye, or a feeling as if the eye was going to burst; and also when the eye, either from its prominence or from a whitish turbid appearance of the cornea, appears to be very much distended.

"The operation is easily performed with the point of a common lancet, or cornea knife, introduced through the cornea into the anterior chamber, at a small distance from its margin; the instrument is then to be turned round a little upon its own axis, so as to allow the fluid to escape more readily by the sides of it. A needle may also be used for the same purpose.

"Other circumstances, besides those mentioned, may demand the puncture of the cornea; as when effusion of blood or purulent matter takes place into the aqueous humor. By this operation the pain from distention may be removed, and bursting of the eyeball prevented." (P. 23.)

Upon the subject of passive or chronic inflammation of the eye, the author is very brief.

*Diseases of the Tunica Conjunctiva.*—Acute inflammation of the conjunctiva is of two kinds: the one consisting of a simple inflammation of the membrane; the other is, in addition, attended with a copious effusion of purulent or muco-purulent matter from the inflamed surface. Hence the one is called *simple,* the other *purulent,* inflammation of the conjunctiva. In the former, the blood-vessels of the membrane are much enlarged, and carry red blood; but unless that part of the conjunctiva which covers the cornea be the chief seat of the disease, or partake in the general inflammation, they never extend over the cornea. The cornea, however, assumes a dim or muddy appearance, which produces more or less indistinctness of vision.

*Of purulent inflammation of the conjunctiva.*—"The purulent or puriform ophthalmia takes place only from some specific cause; as when it occurs symptomatic of small-pox, measles, or erysipelas; or when idiopathic, from infection, as in purulent or Egyptian ophthalmia, gonorrhœa, and infantile purulent ophthalmia. These last probably take place only by the application of morbid virus to the eye. The infantile purulent ophthalmia has been considered to arise from the contact of the matter of fluor albus, imparted from the mother at the time of birth." (P. 34.)

We cannot assert that the infantile purulent ophthalmia is never produced in this manner, although we have observed no facts which appear to support the opinion that the disease does arise from infection thus conveyed. It is certainly not unfrequently occasioned by the tender eyes of the infant being imprudently exposed to a strong light, or by the direct application of spirits to the eye, which nurses almost invariably mix with the water in which the child is washed.

"Those affections of the eye which are called purulent ophthalmiæ, of the different kinds already mentioned, are affections almost entirely of the tunica conjunctiva, from which the pus is copiously secreted. The degree of inflammation which takes place in these puriform affections is in general very great; and the inflammation often spreads to the adjacent parts, so as to occasion abscess or sloughing of the eyelids,—the disorganization of the internal parts of the eye,—opacities, ulceration, or sloughing of the cornea, and suppuration of the eyeball; so that the ravages by these diseases have been truly dreadful, the humors being evacuated in many cases by the bursting of the eye, which then collapses." (P. 34.)

When the inflammation of the conjunctiva is of the purulent kind, the eyelids are so much swollen in the first stage of the disease, that it is often impossible to see the eyeball in a satisfactory manner, even by using a speculum and tearing the eyelids open. "This practice, though recommended by some, may occasion much harm, by the irritation thereby excited." It is of much consequence that the practitioner should bear in mind that minute inspection of the eyeball is not so necessary as to justify any violent measures to obtain it. The violence of the inflammation having subsided, the disease passes into the second, or suppurative stage, and large quantities of purulent matter continue to be discharged for an uncertain period.

"When acute inflammation of the conjunctiva does not terminate by resolution, the most common consequences of it are chronic inflammation, opacity, ulcer, and sloughing of the cornea, with adhesion, prolapsus of the iris, or staphyloma. These, however, are each to be considered as distinct diseases, and treated accordingly."

*Treatment of acute inflammation of the conjunctiva.*—Simple acute inflammation of this membrane generally terminates in resolution. The consequences are sometimes more serious. By its continuation or extension to other parts, vision may be more or less impaired. It, therefore, requires the vigilant and attentive application of the remedies used in acute ophthalmia.

"Great care must be taken to substitute the cold for the warm applications, whenever the violence of the disease is subdued; for, when this is not done, the inflammation is very apt to assume the passive or chronic state, which is generally more difficult to be removed than the active state.

"When the inflammation is of the purulent kind, particular care must be taken frequently to inject the fluids formerly mentioned between the eyelids, and to change the warm for cold applications. In addition to this, it is in general necessary, after washing them, to besmear the edges of the eyelids with some mild ointment, to prevent their adhering together, in consequence of the evaporation of the matter which exudes." (P. 37.)

It is true that these observations have been repeatedly made, but it is equally true that in ordinary practice they are not sufficiently attended to.

*Of passive or chronic inflammation of the conjunctiva.*—This is frequently a most obstinate and tedious form of disease. Inflammation of the conjunctiva may be of a passive or chronic character from its commencement, or it may follow acute inflammation, by gradually passing from the active into the passive state. It varies in degree from a few enlarged vessels upon the eyeball, or inner surface of the eyelids, to the distressing states about to be described.

"1. *Of passive inflammation of the conjunctiva, consisting simply of increased vascularity of the conjunctiva.*—Passive inflammation of the conjunctiva may consist of increased vascularity of the sclerotic part of this membrane, and that which lines the eyelids, without that of the cornea being affected. The vessels have then a deep red colour, inclining to purple, are often tortuous in their course, and have a turgid appearance, without symptoms of acute inflammation being present. In some cases, a rupture of some of these vessels appears to take place, blood being effused beneath the conjunctiva. The conjunctiva has a relaxed appearance, and forms into folds. The patient experiences the sensation of dust or sand existing in the eye, and a feeling of weakness in it.

"2. *Consisting of increased vascularity, accompanied with pustules, or opacity of cornea.*—When passive inflammation of the conjunctiva is not the consequence of acute ophthalmia, it is generally of a pustular character, taking place commonly in subjects of a delicate or irritable constitution. This form of the disease has been denominated strumous ophthalmia. It is most common in young subjects, and is frequently confined only to a small part of the membrane.

"Several distinct and important affections of the eye have been included under the term of scrofulous ophthalmia, from each of these being caused or kept up by that peculiar state of the constitution. The disease, in each of the different forms alluded to, is no doubt modified by the same cause, but it is necessary to distinguish them from each other, in order that the proper treatment may be adopted.

"The different forms in which scrofulous ophthalmia appear are—Symptoms of chronic inflammation of the conjunctiva simply; inflammation of the eye, the same as that last mentioned, attended with opacity, pustule, or ulceration upon the cornea, and in some cases with adhesions and prolapsus of iris; and excoriation, pustule, and ulceration of the edges of the eyelids." (P. 38.)

Strumous ophthalmia most commonly depends upon a debilitated or relaxed and irritable state of the constitution. In its progress, therefore, it undergoes many sudden changes, from the slightest cause. Each of the forms of scrofulous ophthalmia has an acute stage, which, though sometimes of very short duration, differs in no respect from common acute inflammation of the conjunctiva. It is only, therefore, in the second stage, when it becomes chronic, of a distressing and untractable nature, that its true nature is evident.

"The most important and most common form of strumous ophthalmia consists in the inflammation being of the passive kind, having at the same time frequent states of excitement, relapses, or recurrences of the acute inflammation. These are seldom very violent, but frequent, taking place every two or three days, and leaving the vessels of the conjunctiva in an enlarged and relaxed state.

"The passive inflammation is known by the eye having a highly vascular appearance, either over the whole or only a part of it. This increased redness of the eye, though sometimes attended with considerable tenderness to the light and lacrymation, is not accompanied with that pain and heat which would exist in the organ were the inflammation of the acute kind. It also continues for a long time nearly stationary, when proper remedies are not applied.

"When the disease is of the pustular kind, small vesicles or pustules, resembling aphthæ, appear most commonly about the margin of the cornea, sometimes upon the white part of the eye, and at other times, although more rarely, upon the cornea itself. Distinct plexuses of red vessels may, at the same time, be seen upon the conjunctiva, running towards these aphthæ, when there is only one or two; but, when the aphthæ are numerous, and surround the cornea, the increased vascularity upon the white of the eye is then more general. The inflammation which accompanies these pustules, or aphthæ, is generally of the passive kind: the red vessels then appear to be larger, more tortuous, and of a deeper colour than in acute inflammation, and there is no intolerance of light. Sometimes the pustules burst externally, but they may, in general, be removed without doing so." (P. 42.)

The constitutional treatment does not differ, in cases of chronic inflammation of the conjunctiva, from that which is required for the same form of disease when it affects other parts of the eye. As the vessels of the part affected are in a state of relaxation, astringent, stimulant, or escharotic substances will be required as local applications to restore them to a healthy state. The different local remedies enumerated by Mr. Watson are those which are in common use.

Besides the inflammations and their consequences already described, the conjunctiva is often the seat of other diseases of a chronic nature. The most common of these are pterygium and fleshy tumors, or growths upon the conjunctiva. This membrane is also sometimes the seat of cancerous disease.

"A chronic fungous state of the conjunctiva, having somewhat the characters of a cancerous affection, is occasionally met with in the eyes of elderly persons. There are at present before me three cases of this affection, in which it was thought proper to remove the whole of the eyeball along with the diseased conjunctiva. Upon dissection, the coats and other parts seemed to be quite unaffected, and in their natural state." (P. 63.)

The succeeding chapter contains a brief, yet correct, description of the various diseases of the *Cornea.* Upon the subject of acute inflammation of the iris, Mr. Watson observes, in reference to the aversion which some surgeons have to the employment of mercury for its cure, that he has seen many eyes saved by the use of mercury, which otherwise would, in all probability, have been lost, and many lost where this mineral was not used, that might possibly have been saved by this remedy arresting the progress of the disease; for a remission of the disease generally takes place whenever the system becomes affected by the mercury.

*Of acute inflammation of the Retina.*—

"Acute inflammation of the retina is fortunately a disease of rare occurrence, both on account of the violent and distressing symptoms with which it is accompanied, and the complete loss of sight which it often occasions.

"It is attended with the common symptoms of acute ophthalmia, generally to a considerable degree of severity. The symptomatic fever is great. Violent and distracting pain, darting from the bottom of the eyeball through the head, is in general the first and most prominent symptom. The pain comes on suddenly, accompanied with great intolerance of light; the admission of which is compared to a dart passing through the head. The intolerance of light is much increased by moving the eyeball; and is sometimes followed, in the course of a few hours, with total blindness. The patient complains of occasional sparks, vivid flashes of light, and other luminous bodies, appearing before his eyes, both by night and day. Upon inspection, little or no redness is perceived upon the eyeball. The pupil appears in some cases contracted, in others dilated and motionless; the humors to be turbid and muddy.

"In many cases, other parts of the eyeball are affected at the same time with the retina. Along with this disease, therefore, symptoms of inflammation of the conjunctiva, iris, choroid coat, &c. may be present.

"It frequently happens that the symptoms and pain attending inflammation of the retina are so violent, that it resembles inflammation of the brain; the patient being affected, with delirium and want of sleep, to such a degree.

"It need scarcely be remarked, that this is a highly dangerous disease to the organ of vision. It generally terminates with vision being more or less impaired. By the continuance of it, the tunics and humors of the eye become disorganised, so that sight is quite destroyed; and, from the agony which the patients experience, they are often thankful to arrive at a termination to their sufferings, even with the loss of sight.

"The disease is not always fatal to sight. When the proper remedies are applied early, or the disease is only in a slight degree, a perfect recovery may take place. In those cases which I have seen terminate favorably, the eye was long of recovering its wonted vigor. In one case, paralysis of the abductor muscle was the only bad consequence: it continued for some time after the eye recovered. Amaurosis, however, from paralysis of the retina, or its disorganization, is always to be dreaded.

"*Treatment.*—Acute inflammation of the retina demands the employment of the most active remedies. The antiphlogistic treatment should be carried to its greatest extent, to arrest the progress of this violent and destructive disease." (P. 104.)

The chapters on *Amaurosis* and *Cataract* will be consulted with advantage by the student. Mr. Watson remarks, that the arguments for and against the different operations for cataract have certainly been much exaggerated by those who have entered into a discussion of their merit.

"The following results of the operations of several highly eminent surgeons shew the comparative success of the different operations, as well as the success attending operations for cataract generally.

"By extraction, Daviel is said to have operated on $7\frac{1}{12}$ cases successfully to 1 unsuccessfully; but this is doubted. Richter and Sharp, $2\frac{1}{3}$ to 1. In 306 cases operated on by M. Roux, at La Charité, by extraction, the proportion of successful to the unsuccessful cases was $2\frac{1}{3}$ to 1. In 306 cases operated on by M. Dupuytren, at l'Hôtel Dieu, chiefly by depression, the proportion of successful to unsuccessful cases was $5\frac{3}{20}$ to 1.

"Besides the particular kind of operation employed for the cure of cataract, the success depends also on the selection made of the cases and the dexterity of the operator." (P. 157.)

*Of Artificial Pupil.*—Several affections of the eye require the formation of an artificial pupil to restore sight. This operation is necessary when the light is prevented from passing into the interior of the eye, by permanent contraction or closure of the pupil, or by a partial opacity of the cornea.* In such cases, sight can frequently be restored by the formation of a new pupil, or opening in the iris.

"The patient being able to distinguish light from darkness, in cases requiring the formation of artificial pupil, is always a fa-

* Upon this very important operation, much valuable information is contained in Mr. GUTHRIE's elaborate work upon the Operative Surgery of the Eye.

vorable symptom. The absence of it, however, should not be a reason for withholding an operation: for many cases occur where, from the nature of the affection of the iris or cornea, light is prevented from entering to the interior of an eye, which may be otherwise sound.

"On the other hand, there are cases where no reason can be assigned for the patient not being able to distinguish light. There may be a sufficient opening in the pupil, where the direct rays are prevented from passing to the retina by a central opacity of the cornea; so that the patient should be able to see the light, by the rays passing to the retina indirectly by the side of the opacity. The patient should always be able to distinguish light where the pupil is not entirely closed, though it may be so contracted from adhesion to the lens or cornea as to be unfit for distinct sight. In such cases, when the patient does not distinguish light, the prospect of a cure is very uncertain; the case, in all probability, being complicated with amaurosis, or disorganization of the internal parts of the eye: so an operation, though successfully performed, may not restore sight." (P. 163.)

The various cases requiring the operation for artificial pupil are fully described. The manner of performing the operation, of course, varies according to the nature of each particular case.

"The object of each of these operations is the same, being the formation of a new opening in the iris; but this opening may be required either in the centre or at the circumference of the iris; it may be required where the pupil is closed, or where the pupil is in its natural state; and it may be required when the iris adheres either to the cornea or to the capsule of the lens. Some of these operations are performed by simply dividing the iris; others, by the excision of a portion of it; while by others a portion of the iris is detached from its ciliary connexion. By one set of operations, the new opening or pupil is made in the centre of the iris; by another, near to its circumference: so the operator must adapt the operation to each individual case that may come under his care, by selecting the one best suited to its peculiar circumstances.

"Whatever may be the nature of the case, the nearer to the centre of the eye the new pupil can be formed, it will be the more useful to vision. A new pupil has also been found to be the most useful when made at the lower part of the eye. In many cases, however, we have not this in our choice, a small part only of the cornea being transparent, behind which the new pupil must be formed.

"The different modes of performing operations for artificial pupil have been very numerous. The instruments employed, too, have been various and complicated.

"The operations for artificial pupil may be included under three different heads: namely, those operations, 1, by the simple division of the iris; 2, by the excision of a portion of the iris; and, 3d, by the separation of a portion of the iris from its ciliary attachment, either alone or conjoined with the excision of a portion of the iris, or the strangulation of it in an opening made in the cornea." (P. 169.)

In Chapters 10 and 11, a short account is given of *injuries of the Eyeball*, and their treatment, and of *Fungus Hœmatodes* and *Cancer of the Eye.*

Mr. Watson has accomplished in a very creditable manner the task he has undertaken. He has given a concise practical sketch of most of the important diseases of the eye; and, although the work is chiefly designed for students, it will be found a ready practical guide for all who are interested in the subject upon which it treats. The descriptions of the various diseases are rendered very intelligible by the addition of many well-executed plates.

---

*Deafness; its Causes, Prevention, and Cure.* By JOHN STEVENSON, Esq. Member of the Royal College of Surgeons; Lecturer on the Structure, Economy, and Diseases of the Eye and Ear; and Surgeon-Oculist and Aurist Extraordinary to his Royal Highness the Duke of Clarence.—8vo. pp. 262. Colburn, London, 1828.

WE have read this little work with much satisfaction. Its language is brief and explicit, and therefore agreeable and instructive. It is practical, but in a general way, without descending to those minute details of particular treatment said to possess panacean efficacy, in which quackery delights to revel. Yet it is well adapted for the study of junior members of the profession, and those scientific *dilettanti* whose state of health contributes to render them interested in its subject. We believe it is chiefly with a view to the instruction and benefit of such readers that the author has prefixed a simple and interesting description of the anatomy of the less intricate parts of the ear, and has wisely forborne, in so elementary a treatise, to lead his tyros into the labyrinth, where, without the conducting thread of minute anatomy, they would be entirely lost.

Adopting the method of VALSALVA in his description of the ear, he divides it into external, middle, and internal portions; and, in his history of the shape of these different parts, he successfully calls in the aid of comparative anatomy to enable him more completely and clearly to point out and illustrate the design and use for which each particular part was intended. In doing this he has given additional interest to his work, and thus, by keeping up the attention of the reader, has, while assisting the judgment to comprehend, enabled the memory also to retain the facts. We may quote the following passage as an example:

"With regard to the structure of the passage into the ear, there are several singular varieties observable in different animals. In the owl, for example, which for the most part perches on a branch of a tree, and hearkens after the prey *beneath* her, it is produced further out *above* than below, for the better reception of the least sound. But in the fox, which prowls underneath his prey at roost, it projects more *below* than above. In the polecat, an animal that listens straightforward, it is produced *behind*. In the timid hare, an animal possessed of an exceedingly acute sense of hearing, and whose care it is to avoid her pursuers, it is furnished with a bony tube, which, as a natural otocoustic, is so directed *backward* as to receive the smallest and most distant sound that comes *behind* her." (P. 25.)

The author, in speaking of the membrane of the drum, concludes that it presents no natural aperture; and we think with good reason, from the following experiment: "If quicksilver be poured into the meatus, it will not pass into the tympanum; nor, if injected through the eustachian tube, will it find its way into the outer passage: a clear demonstration of the perfection and entireness of the membrane." He tells us, that "it was formerly supposed by Rivinus that there is a natural aperture through the membrane of the drum." We are surprised he does not mention the names of Vest and Wittman, who have so recently maintained the same opinion.

Mr. Stevenson relates that some physiologists have attributed to the eustachian tube the office of conveying sound, but he does not say that such is his own opinion. We are firmly persuaded that this is an erroneous idea, and that nature intended that this tube should serve only to renew the air of the tympanum when requisite, and in that way alone promote the perception of sound: and we ground our opinion upon the simple experiment of stopping the ears of an individual, and then attempting to make him hear by speaking into his mouth. Indeed, the fact of our opening our mouth when we listen with great attention, and the assistance we seem to derive from this act in perceiving sounds, is easily explained without having recourse to the eustachian tubes; since every anatomist is aware that the depression of the inferior maxilla, in opening the mouth, causes its condyles, which are situated anterior to the meatus auditorii externi, to descend and to be carried forward, so that they become somewhat dilated; which is evident to any one introducing a finger into the ear while opening his mouth.

After dismissing the anatomy of the ear, the author enters upon its physiology, and the nature of sound; in which part of his work he has assembled some striking facts and experiments, collected from the wide and fruitful fields of acoustics and comparative anatomy. But they are too much interwoven with the thread of his subject to admit of partial quotation.

We consider the following fact of practical utility, but we are averse from believing the cranium a *sensible* solid, in the sense of Mr. CLOUGH; since it is well known that all solid bodies convey sounds better than air, water, or such like thin media.

"Mr. Clough, late of Manchester, has made some ingenious experiments, from which he infers that the cranium, or skull, is a really sensible solid. We know, indeed, that a watch held betwixt the teeth, or even applied to the head, can be heard by a person who is deaf to impressions conveyed through the air. It is partly in this way that we can judge whether deafness may be cured by an operation, as depending on some injury of the mechanism of the organ, or whether it be an incurable affection of the nerve or brain itself. For, if the sound be perceptible when conveyed through the teeth, or when a watch is pressed against the mastoid process, we are assured that the internal organ is unaffected; which assurance may lead us to detect the seat of the disease to be either in the outer passage of the ear, the drum, or the eustachian tube, and to regulate our measures accordingly." (P. 72.)

By way of preface to the practical part of this treatise, the author contrasts the slight attention which the ear, though a highly complex and important organ, has received, with that which the profession has so liberally and "successfully" bestowed on the organ of sight. He suggests that this neglect of the profession is attributable to these three causes: first, "the frequent unsatisfactory result" of the treatment of its diseases; secondly, "the difficulty of ascertaining the more deep-seated morbid affections, owing to the inaccessibility of the interior parts of the ear;" thirdly, "the circumstance of the diseases themselves scarcely ever proving fatal, which precludes accurate examination after death; and seldom producing pain, the great monitor of disease, during their existence." Now it appears to us that the author has mentioned, in his two first heads, what is as nearly applicable to the "sister organ." The third is certainly equally true in regard to some of the best understood and most afflicting diseases of the eye. And if it be true, which we doubt, that the diseases of the ear are, as the author affirms, so much less under our control, we should be more inclined to attribute our ignorance and darkness in this particular to the greater value and preference we naturally give to the perception of light, compared with that of sound; and, though the ear be very precious to us, and of high utility, and a great provider of pleasure, yet the eye has still nobler and more important functions: it delights in perusing "the human face divine;" with its rapid glance it reaches the heavens, measures and numbers the "pensile planets," and is equally competent to examine and admire the most minute and delicate beauties of nature. Blessed with a perfect eye, the deaf are almost as independent as other men, and, like Ajax of old, need only pray for light. If the ear can collect grateful sounds, as the eye can contemplate pleasing objects, it cannot assist to raise the mind by magnificent and exalted ideas. It imparts the luxuries of speech; it conveys the charms of music; but still it is decidedly inferior to the eye in keeping up our connexion with the external world. There is, we conceive, another reason why it might be supposed that the diseases of the ear could be cultivated with less zeal and less widely than ocular maladies: we allude to the greater facility with which the small number of operations required in these complaints may be performed; for we conceive (*cæteris paribus*), paradoxical as it may appear to some minds, that, in proportion to medical or surgical difficulties presented by any disease, is the degree of professional industry and talents which are employed to overcome them. This opinion is supported by merely referring to the long list of names enrolled against phthisis, tetanus, and cancer.

The author very judiciously warns the unprofessional readers not to imagine themselves capable, immediately on perusing his publication, of treating their own complaints; and tells us he has designedly endeavoured to render his work ill calculated to assist them in so dangerous a purpose. This advice is not uncalled for at the present time, when conceit and quackery are predominant.

Mr. Stevenson's method of treating the diseases of the ear is simple and scientific. He is careful throughout to recommend a strict attention to the constitutional indications while combating the local ailments. This part of his work, commencing with the diseases of the auricle, and conducting our research inwards, concludes with a description of those of the labyrinth and *nervous* deafness.

The following is his account of herpetic eruption of the auricle, which complaint, from bad management, is apt to have very serious consequence, as the annexed brief case fully shows:

"~~But~~ the most troublesome and distressing ailment to which the auricle is liable is an herpetic eruption, consisting of numerous small watery pimples, or vesicles, surrounded by an inflamed base. These little vesicles bursting spontaneously, or being more frequently ruptured by the fingers of the patient, who is almost irresistibly impelled to rub or scratch them, with a view to allay the accompanying almost intolerable smarting and itching, they pour out a copious discharge, which soon becoming fetid and acrimonious, occasions irritation, excoriation, and often ulceration of the affected surface.

"If the progress of this disease be not speedily arrested, the skin and subjacent cellular texture begin to thicken and enlarge to such a degree as to render the auricle, already inflamed, disgustingly frightful and deformed. Nor is this the termination of the mischief. In consequence of the tumefaction extending to the soft parts of the auditory canal, and of the inspissation of the discharge, the area of this tube becomes so much narrowed, and in some instances so completely obliterated, as to offer a considerable barrier, or total obstruction, to the ingress of sound, causing, while the disorder continues, either partial or total deafness.

"Some time since, I was consulted by the daughter of a nobleman, who had suffered for eighteen years from the protracted obstinacy of this disease. By constitutional as well as local remedies, adapted to the nature and urgency of the symptoms, they gradually subsided: the auricle regained its healthy appearance and proper size; and the function of the ear, which had been so long suspended, was at the same time completely restored." (P. 124.)

This is followed by several other interesting cases, which we of course cannot quote. Mr. Stevenson, after describing the leading symptoms of disease in these cases, should have at least *named* the remedies he employed, and should not, in our opinion, have contented himself with merely saying, "Alterative and constitutional, combined with topical, remedies were employed;" especially as he affirms in the adjoining paragraph, "the part particularly affected likewise demands the most *careful* and *appropriate management.*" x

Under the article "*diseases of the outer passage of the ear,*" the author remarks, that the earache is often occasioned by the present fashion of wearing the hair, in doing homage to which we are "stripped of the pendant *side locks,* the real ornaments and guardians of the ear."

"Nature, unsophisticated nature, (by which I mean the Creative x This reason is, as mentioned before, that he wants folks to cure themselves.

Power,) does nothing in vain. Is there no utility, to say nothing of beauty, arising from the partial concealment of the auricle by unrestrained tresses waving from the temples, and hanging gracefully by the side of the face? Such a distribution of the hair not only protects the ear from the intrusion of winged insects and light substances which move in the liquid firmament, but likewise, by breaking the force of cold winds, guards the organ from the dangerous influence of atmospheric changes.

"That such is the natural office and effect of the hair, through the interstices of which the undulations of sound readily penetrate and gain admission into the auditory passage, may be inferred from the well-known analogous fact of a thin net veil affording a salutary shelter or guard against the rude assaults of the wintry blast.

"The removal of the side locks, by exposing the ear to the partial application of cold air, becomes a fruitful source of deafness, originally induced by inflammation of the passage, and consequent suspended secretion of wax. Accordingly the hairdressers even warn the profession on this point: one of the most respectable of them informs me, that, since this fashion has been in vogue, many of his customers complain to him of pains in the ear, and increasing difficulty in the function of hearing; doubtless attributable to what may be justly termed a mutilation of the elegant shelter ordained by nature for this important organ." (P. 135.)

He also inveighs against the use of "nightcaps made of flannel, thick cotton, or dense silk," and considers the complete desertion of them an advantage; and adds, that, by proscribing them, he has cured many of his patients and friends of "that oppressive weight and morning headache so often complained of." The author here furnishes us with a theory of the manner in which the cap becomes injurious, which is very plausible, and may be true; and, in confirmation of his practice, we may relate that we know more than one individual who lost, with the habit of wearing nightcaps, their tendency to nasal colds and occasional earache.

In section 5, on "*extraneous substances or insects in the outer ear*," and in section 6, "*redundancy or deficiency of wax*," the author more than once reprobates the *injudicious* or unskilful use of instruments, by which he informs us the membrane is sometimes ruptured, and otherwise injured. We believe ear-pickers are not so much in vogue as formerly, and it would be well for the public did they entirely forego the use of them, if the opinion of a great physician of the eighteenth century was correct. Our author says,

"So firmly persuaded was Sir Hans Sloane of the bad effects of these instruments, that, in a paper which he wrote on the subject in the Philosophical Transactions, he does not hesitate to declare that he could trace to their officious use nearly all the cases of deafness which were brought for his assistance." (P. 166.)

The distressing consequences caused by a foreign body in the ear is well exemplified in the following case. We learn that the subject of it was an "officer of high rank and distinguished courage during the Peninsular war." He had been wounded, and, while only convalescent, his steed propelled him against the branches of an oak, which lacerated the auricle. "External inflammation of the ear was thus produced, which was succeeded by a copious purulent discharge.

"In spite of the various applications resorted to with a view to suppress the disease, matter continued to issue from the outer passage in considerable quantity.

"Returning to England in this state, and with the sense of hearing nearly extinguished, he called to consult me on the subject. Upon attentively inspecting the tube in a clear light, I noticed something lying at the very bottom of the passage, which, on cautiously pressing upon it with a probe, proved to be hard and resisting, but at the same time slightly moveable.

"On considering the concussion he had sustained, in connexion with all the other circumstances of the case, I was led to suspect that a portion of the bony meatus, being fractured, had become detached, and by its presence served to keep up the discharge.

"I proposed, at all events, whatever might be its nature, to extract this body; an operation which, with some difficulty, was accomplished. On examining the extraneous substance, after freeing it from all adhesive matter, I found that it was a splinter of oak, the introduction of which could easily be explained, nearly half an inch in length, and about two lines in breadth, one pointed extremity whereof had penetrated through the membrane of the drum, whilst the remainder lay fixed across the passage in the angle formed at its farther extremity.

"This foreign substance having been removed, the ulcer which it produced and kept in a perpetual state of irritation, speedily healed, and the hearing was perfectly restored." (P. 157.)

We shall not enumerate the various means the author recommends for the extraction of foreign substances and insects from the ear; but we consider his remarks on this subject, as well as elsewhere, those of a person who has had long practice and experience. It is curious that Celsus should, after advising many rational remedies and modes for the removing from the ear insects and other substances, conclude by recommending this unlikely and barbarous plan, which we insert to shew the futility of talents, if correct anatomical knowledge is wanting. "Tabula quoque collocatur media inhaerens, capitibus utrinque pendentibus, superque eam homo deligatur in id latus versus, cujus auris eo modo laborat, sic, ut extra tabulam non emineat; tum malleo caput tabulæ, quod a pedibus est feritur: atque ita concussa aure, id quod inest, excidit."

Mr. Stevenson gives the following signs for detecting the imperviousness of the eustachian tube:

"The obstruction or obliteration of this tube may be suspected as the cause of deafness, by ascertaining whether any syphilitic ulcer, sloughing putrid sorethroat (cynanche maligna), enlarged tonsils, or descent of a nasal polypus, has preceded the disease. Further information may likewise be gained by causing the patient to inflate the tympanum: if he possesses this power, the duct must be free; but the converse does not necessarily follow, since all have not the tact and ability to force air into the cavity of the drum, who may nevertheless have a pervious eustachian tube. We have not, indeed, any infallible criteria by which to judge of the existence of this disease, the whole of our knowledge on the subject amounting only to what may be called presumptive evidence." (P. 214.)

"The eustachian trumpet," says our author, "may be closed either in the bony portion of the tube, by ossification, which comes on gradually, and without any premonitory symptoms; or, what is far more frequent, by an obstruction of the mouth of the duct, which opens at the side of the throat. If, therefore, a patient complain of deafness unattended with other symptoms, such as noises in the head, or any of those sensations which indicate a deranged state of the auditory nerve, and we find, upon inquiry, that he has previously laboured under, or is actually suffering, either of the local affections above mentioned; and, further, that he cannot, by filling the mouth with air, force it into the tympanum, it is reasonable to infer that an obstruction of the eustachian tube is the true cause and essence of the disease.

"But simple inflammation of this tube more frequently produces deafness than is generally supposed; the nature and symptoms of which, though exceedingly distressing, and sometimes even alarming, not uncommonly escape detection. In illustration of this, I mention the following cases.

"A highly respectable lady wrote to me, some months since, respecting her daughter, about nineteen years of age, who laboured under a total deafness in one ear, and a great defect of hearing in the other.

"She represented the disease to have come on gradually with a slight cold, sore throat, and hoarseness; symptoms which were soon associated with occasional shooting pains in the side of the neck, extending to the back part and side of the head. These attacked her by paroxysms, the violence of which increased so much as at times to induce fainting. Her nights were restless, her appetite and digestion much impaired, and she became in consequence exceedingly weak and emaciated. Having at the same time cough and purulent expectoration in the morning, with a white tongue, quick pulse, and general febrile irritation, it was feared that the case would terminate in consumption of the lungs, (phthisis pulmonalis.)

"The symptoms were too complex and formidable to warrant my venturing to prescribe without the opportunity of a personal inquiry into all the circumstances of the case, which being declared by her justly eminent medical attendants to be highly discouraging, she determined, although residing at a distance of near two hundred miles, to hazard, by easy stages, a journey to London, in order that she might place herself under my immediate care. With great difficulty and some danger, her object was accomplished; and, after a full investigation of the symptoms, I satisfied myself that they originated from an inflammation of the eustachian tube, the guttural extremity of which having become slightly ulcerated, afforded the purulent expectoration.

"With this conviction on my mind, I prescribed external irritants, and the local application of fumigations and gargles to the inflamed and ulcerated surface, together with appropriate constitutional treatment: the disease was thus gradually subdued, and I had the gratification of restoring my patient not only to perfect health, but also to the full enjoyment of her hearing." (P. 216.)

This case is followed by others of similar importance.

The French speak with more certainty on this subject, and place great reliance on the annexed experiment. Having made the patient lie on one of his sides, and then filled the meatus auditorius externus of the opposite ear with water, they direct him to make several powerful expirations; when, if the tube is pervious, it will be indicated by the alternate rise and fall of the fluid in the ear, corresponding with the action of the lungs.

These are our author's directions for puncturing the drum where the tube is permanently obliterated.

"Preparatory to commencing this operation, the patient must be placed in a strong light, and in such a position that the passage may be fully illuminated, and the membrane rendered plainly visible. A small triangular-pointed trocar, made with a shoulder for this especial purpose, or the pointed end of a common silver probe, —which, in default of the former instrument, I have used with success,—must be carefully thrust through the anterior and lower part of the membrana tympani. Great caution is requisite, in order to avoid touching the handle of the malleus, which might, with its articulated chain of bones, be dislocated from their connexion; an accident which would irreparably injure the function of the organ.

"It is also necessary to take care that the instrument be not allowed to penetrate too far into the cavity of the tympanum, lest its vascular lining be wounded; in which event blood being effused and coagulating, might become organised, and render nugatory the effect of the operation.

"A cracking noise will be instantly perceived on the puncture being made, similar to what is occasioned by the pricking of thin parchment whilst stretched, which is more loud and sharp if the tube be totally obstructed, from the rapid entrance of air through the small aperture. The patient, if his case be adapted to this mode of treatment, is instantaneously restored to hearing.

"The object and effect of the process is, the substitution of the artificial small hole in the membrana tympani for the obstructed eustachian tube, by which the air being again admitted into the cavity of the drum, the mobility of the membrane returns, and the action of the small bones, and all the connecting machinery, is to a certain extent re-established.

"The puncture is, however, apt to close, as Valsalva found in his experiments on a dog, and occasionally requires to be repeated two or three times before the aperture, by being made fistulous, becomes patulous and open.

"In one instance,—that of a respectable female who resided in St. Paul's Church yard, whose hearing I succeeded in restoring by this method,—I had occasion to introduce the trocar a second time, in consequence of a reunion of the puncture, and return of deafness; after which, however, it remained permanently open, and the function of the organ continued complete.

"This operation, though apparently simple in the hands of an expert practitioner, requires so accurate a knowledge of the situation and structure of the parts to be acted on, and of the manner of introducing the instrument with success, and without causing mischief, that it ought never to be undertaken by any but such as are qualified for the task by anatomical knowledge and surgical skill." (P. 230.)

We may here remark, that Andral advises us to introduce the extremity of a "*sonde cannelée*" into the puncture every other day during the two first weeks, in order to prevent the adhesion of its sides.

The author does not state the average success he has experienced in this operation; but we know that the celebrated Itard relates that he only succeeded twice in ten operations of this kind for the cure of deafness.

Deleau has several times, he informs us, injected air through the eustachian tube, with great advantage, in certain cases of deafness; so that, through these "*douches d'air,*" as he calls them, patients, who for years could not hear a watch at the distance of a few inches, have, after a moderate trial of this remedy, been able to hear the same sound several feet from them. We wish the author had given us his opinion of this remedy. It is singular that injecting through the eustachian tube was, in France, first practised by a post-master of Versailles, who lived during the last century, and performed the operation on himself!

We have now brought our review of this work to a close; but having already expressed our opinion of its merits in detail as we passed through its different divisions, we have nothing more to add in conclusion, than that it may safely be consulted as a book of reference by either the student or practitioner.

Perhaps, Mr. Stevenson will excuse our suggesting to him that the detail of a case can never be made additionally interesting by the information that the patient resided "in one of our fashionable squares," or that he was "an officer of high rank and distinguished courage during the Peninsular war."

---

*Pathological and Surgical Observations relating to Injuries of the Brain.* By B. C. BRODIE, F.R.S. Surgeon to St. George's Hospital. Part I. [From the *Medico-Chirurgical Transactions*, vol. xiv.]

ALTHOUTH much valuable information upon this subject lies scattered throughout the mass of surgical literature, no practical writer, with whose works Mr. Brodie is acquainted, has attempted to make such an arrangement and collection of facts as will enable the surgical student to take a distinct and connected view of all the parts of this interesting inquiry.

Mr. Brodie first gives an account of the immediate effects of injuries of the head as indicated by dissection; and in the next section he treats "*on Concussion of the Brain.*" Many of the consequences of an injury of the head which are disclosed to us by dissection, are not likely to be marked by any peculiar symptoms in the living person, at least not previous to the access of inflammation. It has been long established, that another cause besides those detected upon dissection may be concerned in producing the symptoms which immediately follow a contusion of the head.

"A man receives a blow on the head; he becomes insensible, and continues so for a few minutes, or for several hours. He dies, in consequence of this or some other injury; and, on examination after death, the brain and its coverings appear to be perfect in all their parts; so that the most accurate anatomist can discover nothing different from the natural appearance of these organs. Opportunities of verifying this observation occur more or less to all those who have had much experience in their profession. In such cases, the patient is said to have been stunned, or to have suffered from concussion of the brain: and it is to one of these three causes, namely, concussion, compression, and wounds of the brain, that the symptoms which immediately follow an injury of the head, and which are antecedent to those produced by inflammation, are to be referred.

"Opportunities of inspecting the brain, where the patient has laboured under symptoms of concussion, may arise, first, where the concussion has so disturbed the functions of that organ as to have been in itself a cause of death (which is, on the whole, a rare occurrence). Secondly, where the concussion of the brain has been complicated with other and still more serious mischief. We learn from such examinations, that the symptoms which are ascribed to concussion do not depend on any such derangement of the organization as admits of being disclosed to us by dissection. The brain appears to retain its natural structure unimpaired. We are not, however, justified in the conclusion that there is therefore in reality no organic injury. It is difficult to conceive in what other manner concussion of the brain can operate so as to produce the effects which it is known to produce; and if we consider that the ultimate structüre of the brain is on so minute a scale that our senses are incapable of detecting it, it is evident that there may be changes and alterations of structure, which our senses are incapable of detecting also. The speedy subsiding of the symptoms of concussion does not contradict this opinion. A deep incised wound in other parts of the body may, under certain circumstances, be completely and firmly united in the space of twenty-four hours; and it is easy to suppose that the effects of a much slighter injury may be repaired in a still shorter space of time.

"The disturbance of the functions of the brain, which is the consequence of concussion, may exist in various degrees, and may be of various duration.

"In many instances there is at first complete insensibility to external impressions. The patient lies as if in a state of apoplexy, from which, however, he recovers in the course of a few minutes. In some instances the recovery is complete; the patient rises and walks away as if nothing unusual had occurred. In other cases, this state of total insensibility is followed by one in which the sensibility is impaired, but not destroyed. The patient is not affected by ordinary impressions, but, if spoken to in a loud tone of voice, he will shift his position, and answer in a peevish manner. Sometimes he is in a state of imperfect delirium, talking in an incoherent and rambling manner, as if intoxicated. The pupils contract on exposure to light, and are sometimes more contracted than under ordinary circumstances. There is no paralysis. The respiration, in the great majority of cases, is performed easily and naturally; in a few instances only it is laboured, and approaching to being stertorous. These symptoms may wholly subside in the course of a few hours, or they may continue for three or four days. In the latter case it frequently occurs that the patient regains his sensibility for a time, and then relapses into his former condition. Where inflammation of the brain follows the injury done by concussion, it may be that the primary effects of the concussion are entirely relieved, so that there is a considerable interval of sense before the inflammation shews itself. But it may be also that there is no such interval; and the symptoms of concussion, in this last case, are gradually and imperceptibly converted into those of inflammation.

"Concussion of the brain, in almost every instance, occasions headache; sometimes a slight headache, which is speedily relieved; at other times an intense headache, which may remain for some days, a solitary symptom, after all other symptoms are vanished. Sickness and vomiting for the most part are early symptoms, and seldom continue after the patient has recovered from the first shock of the accident. Of course, there is no recollection afterwards of what occurred during the period of complete insensibility. The memory, however, is sometimes affected to a still greater extent; and the impressions made on the mind by the events immediately antecedent to the injury become obliterated. A groom in the employment of the Persian ambassador, in the summer of 1819, was engaged in cleaning one of the ambassador's horses, when he received a kick from the animal on the head. He did not fall, nor was he actually insensible or stunned; but he entirely forgot in what employment he had been engaged at the time of receiving the blow. Being unable to account for the time which had elapsed, he concluded that he had been asleep: said so to his fellow-servants, observing at the same time that 'he must set to work to clean the horse, which he ought to have done before, instead of going to sleep.'—A boy going down into the hold of a ship fell from a considerable height, and struck his head. He lay insensible, as it appeared from the observation of his shipmates, about half an hour, when he came upon deck without any assistance. Nevertheless, on the following day, all the circumstances of the accident had passed from his memory. Some time afterwards, when he was received into St. George's Hospital, I found that he knew nothing of the accident except from the report of others. He had not only entirely forgotten the accident itself, but he did not even remember his having gone down into the hold of the vessel before the accident, nor his having come upon deck afterwards; and he never regained his recollection on these points. Desault mentions the case of a man, who, after a blow on the head, at first had no recollection except of recent events; but afterwards a change took place, in consequence of which his memory failed him as to recent events, while he could remember those which had occurred in childhood.

"A number of circumstances which it is unnecessary to enumerate, as every physiologist is well acquainted with them, tend to shew that the influence of the brain is by no means necessary to the action of the heart; which may, under certain circumstances, continue uninterrupted, even after the entire removal of the head. Nevertheless, in cases of concussion of the brain, we generally find the circulation more or less affected; the pulse intermitting, irregular, feeble, perhaps scarcely perceptible, and the patient in a state approaching to that of syncope; and such may be his condition for a few minutes, or for the first four or five hours after the infliction of the injury. The connexion and sympathy which exist between the different parts of the nervous system, afford a reasonable explanation of this apparent anomaly, which, however remarkable it may be, is not more remarkable than the syncope which not unfrequently follows the first introduction of a bougie into the urethra, or that which is the consequence of many other trifling injuries of parts remote from the centre of the circulation, and exercising no direct influence over the functions of the heart.

"In those cases in which concussion proves fatal, it appears to be this disturbance of the heart's action which is the immediate cause of death. In general, when the patient has lain for some time in the state which has been described, a reaction of the circulating system takes place, and the pulse beats with greater strength in proportion as the failure of it was greater in the first instance. But where the shock has been unusually severe, there is no such reaction. The pulse becomes more and more feeble, more irregular and intermittent; the extremities grow cold; and at last, the action of the heart being altogether suspended, the patient expires. In some cases, even after reaction has begun to take place, it seems as if the constitution was unequal to the effort: there is another failure of the circulation, the result of which is the same as if the patient had never rallied from the beginning.

"Sect. 4. *Compression of the Brain.*—If the dimensions of the cavity of the cranium be suddenly diminished, as in a case of fracture with depression of bone, or if the actual quantity of the contents of the cranium be increased, as in a case of ruptured vessel and extravasation of blood, the functions of the brain become impaired. This is a matter of experience and observation, about which there is no dispute. There may be, however, some difference of opinion as to the physiological explanation of the phenomena which arise in such cases. It has been usually held that the substance of the brain is actually compressed; but Mr. Bell observes, very truly, that we have no more right to believe that the substance of the brain admits of being compressed, than that water is compressible; and he infers that what is called compression of the brain operates not on the substance of the brain itself, but simply on its blood-vessels; lessening their diameter, and thus preventing that due supply of scarlet arterial blood which is necessary to a due performance of the vital functions. It is evident, indeed, that the effect which compression of the brain produces on its vessels must be to a greater or less extent, such as Mr. Bell has described it to be. It may, however, be urged on the other hand, first, that in some cases symptoms similar to those which arise from compression take place where there is a preternatural determination of blood to the head; where the vessels, instead of being empty, are actually overloaded; and that in these cases the symptoms are relieved by drawing blood from the jugular vein, or from the veins of the arm; as if the pressure occasioned by too much blood in the vessels was productive of nearly the same effects on the brain with that arising from blood in a state of extravasation; secondly, that, although we admit the substance of the brain to be incapable of being compressed into a smaller compass, yet that the effect of all pressure on it must be, and is, to alter the position and relative situation of the delicate fibres of which its minute structure is composed, and that we need seek no further explanation of the symptoms which are met with in these cases.

"In whatever way compression of the brain operates so as to disturb the functions of that organ, it is difficult to explain wherefore the symptoms to which it gives rise are sometimes slight, and at other times urgent, although occurring under circumstances apparently similar. A depression of bone, which in one instance produces comparatively little effect, in another case occasions a manifest destruction of sensibility: and the same observation may be made respecting internal extravasations of blood. Every practical surgeon must have observed that there are differences in the symptoms produced, which are not to be accounted for by any difference in the quantity of pressure, nor in the particular part of the brain which is affected by it. At the same time it is undoubtedly true that, for the most part, the patient suffers more from an extensive than from a slight depression; more from a large than from a small extravasation. There is reason to believe that pressure is, on the whole, more dangerous when it affects the lower part of the brain, than when it affects the upper part; and it has appeared to me that more urgent symptoms are produced by a given quantity of blood, when it is effused into the cells between the tunica arachnoides and pia mater, than when it is collected in one mass so as to produce a less general pressure."

Mr. Brodie next proceeds to consider the particular symptoms which arise from pressure on the brain. He is of opinion that there is not any such difference in the character of the insensibility produced by concussion and that produced by compression of the brain, as will enable us at once, and in all cases, to distinguish these two kinds of injury from each other. For example:

"A woman received a blow on the head; after which she was able to walk home, complaining that her head was hurt, and that she had received her death blow. In an hour after the accident, she gradually became insensible. About fourteen hours afterwards she was brought to St. George's Hospital, labouring under symptoms precisely corresponding to those which have been described by Mr. Abernethy as arising from concussion. These symptoms continued, and even rather abated than increased, until the third day, when an aggravation of them took place, and she expired. On examining the body, eight ounces of blood were found effused underneath the dura mater. The circumstance of there having been no loss of sense in the first instance, and the interval of an hour which elapsed between the period of the accident and that of the occurrence of the symptoms, sufficiently demonstrate that they were the consequence of pressure produced by the hemorrhage, and not of the concussion."

In some cases sensibility is destroyed in one part of the system, while the general sensibility is but slightly impaired. Mr. Brodie has never met with an instance, in cases of hemiplegia after an injury of the head, in which the paralysis was not on the side opposite to that on which the pressure existed. This observation, however, does not apply to more partial paralytic affections. The state of the pupils varies very much in cases of pressure on the brain, even under circumstances apparently similar. The author has seen the pupils dilate with the absence, and contract with the presence of light, although the patient lay in a state of complete insensibility. Generally, however, where the other symptoms of pressure are present, the pupils are insensible and motionless, being usually dilated, but sometimes contracted. Sometimes the pupils remain dilated for a time, then contract suddenly, and again dilate; these changes taking place independently of light and darkness. Mr. B. has observed, especially where the pupils have been dilated, that they frequently contract immediately after bleeding; the dilatation returning when the immediate effect of the bleeding has ceased. Dr. Hennen mentions a case in which blood was extravasated among the membranes of the brain, and in which the pupils were observed sometimes to dilate with an increase, and to contract with a diminution, of light. We have frequently observed the same fact when examining the eyes of children either during the existence of convulsions, or previous to the occurrence of the paroxysm, when the symptoms indicated its approach. One pupil may be dilated while the other is contracted.

Does secondary hemorrhage, Mr. Brodie asks, ever occur within the cavity of the cranium? Such an occurrence is thought to be very rare, but it probably happened in the following case:

"A man, thirty-five years of age, on the afternoon of the 8th of November, fell from a cart, and struck his head against the pavement. A medical practitioner in the neighbourhood bled him, and he was afterwards brought to St. George's Hospital, talking and reeling like a drunken man. He was again bled. On the following day he complained of headache, but was otherwise well. He continued without any symptoms until five in the morning of the 12th of November, when some of the patients in the same ward heard him talking incoherently. The nurse called the house surgeon to him, but before he could arrive the man had become insensible, and was found lying motionless, with stertorous respiration and dilated pupils. Blood was taken from the arm, but the symptoms were not relieved, and he died in about half an hour after the commencement of the attack. On examining the contents of the cranium after death, a thin layer of blood was found extravasated in the cells between the tunica arachnoides and the pia mater, where those membranes cover the posterior part of the two hemispheres of the cerebrum. In the lower part of the right anterior lobe of the cerebrum, the substance of the brain had been ruptured; and underneath this part, between the dura mater and tunica arachnoides, there was a collection of about two ounces and a half of blood. This last had all the appearance of a recent extravasation, and seemed to afford a satisfactory explanation of the sudden alteration in the symptoms which immediately preceded the patient's dissolution: the hemorrhage in the first instance having in all probability been checked by the blood-letting which was resorted to both immediately after the accident and on his admission into the hospital."

The peculiar danger of wounds of the brain arises, in the great majority of instances, not from the immediate effects of the injury, but from the extensive and intractable inflammation which takes place afterwards.

*Treatment of concussion of the brain.*—It is commonly remarked that two opposite modes of treatment have been recommended in cases of concussion of the brain: stimulants and cordials; bleeding and antiphlogistic remedies. Mr. Brodie remarks, that the opposition of opinion is greater in appearance than in reality.

"I am inclined to believe that, if the advocates of the respective systems were questioned on the subject, it would be found that the views which they entertain are not essentially dissimilar. I suppose that none of those who have suggested the exhibition of stimulants would actually be inclined to apply this practice to cases in which the pulse has regained its strength and regularity; and, on the other hand, I conclude that no one among those who have advised the use of the lancet would think of taking away blood when the patient lies with pale cheeks and cold extremities, and a feeble and intermitting pulse, or would refuse to resort to the cautious exhibition of cordials and stimulants where these symptoms are so urgent that he is manifestly in danger of sinking, in consequence of the depressed state of the circulation which has followed the first shock of the injury.

"Cases of this last description are, however, in reality of rare occurrence; and there are, indeed, sufficient reasons why we should regard that condition of the system which approaches to syncope as being, in the great majority of instances in which it exists, conducive to the patient's welfare, and why we should wish to prolong, rather than to abridge, the period of its duration. The same blow which gives rise to symptoms of concussion frequently occasions the rupture of some small vessels within the cranium. The same state of the system which produces an enfeebled action of the heart, is calculated to prevent the ruptured vessels from pouring out their contents; and, the longer it continues, the less is the danger of internal hemorrhage. If we artificially excite the action of the heart by the exhibition of wine and ammonia, we are in danger of inducing symptoms of pressure on the brain. If, on the contrary, we watch the gradual restoration of the pulse, and at the proper moment take from the arm a sufficient quantity of blood to prevent the heart resuming its wonted action, it is probable that we may often succeed in checking or arresting an extravasation of blood on the surface of the brain, or among its membranes, which might otherwise prove fatal. There is also the following very important circumstance, which is not to be overlooked in this part of the inquiry. A state of depression is followed by a state of excitement. As the patient recovers from the former, the pulse, with respect to fulness and strength, becomes raised above the natural standard; and it is evident that this affords an additional argument in favor of the practice which is here recommended.

"The same views respecting the prevention of internal hemorrhage which incline us to take blood from the arm in the first instance, cannot fail to influence our conduct afterwards. There is no evident reason why vessels, which have once bled, should not be liable to bleed again within the cranium, as well as in other situations. I have already mentioned a case in which a patient, who was apparently going on favorably, suddenly expired in consequence of such secondary hemorrhage, on the fourth day after the occurrence of the injury. If similar cases are rare, this may reasonably be attributed to the remedies which modern surgeons, with few exceptions, do not fail to employ. At any rate, where so much is at stake, we are called upon to neglect no measures of precaution; and, however small the danger from this cause may really be, the surgeon should provide against it, by frequently inquiring into the state of his patient; by urging the necessity of continued repose of body and mind, by limiting him to a scanty vegetable diet, by the exhibition of laxative medicine, and by the abstraction of blood, whenever the state of the pulse indicates that this may be done with propriety.

"Independently of the foregoing, there are other considerations, which might of themselves lead us to adopt the same method of treatment. I believe that the patient, in cases of concussion, will generally spontaneously recover from that state of insensibility in which he remains after the vigor of the circulation is restored. But, nevertheless, from the best observations which I have made on the subject, I cannot doubt that his recovery is much assisted by repose and low diet, and depleting remedies. Often, immediately after being bled, the patient, who before was in a state of stupor, exhibits manifest signs of returning sense. Further, it may be urged that concussion is liable to be followed by inflammation of the brain, or its membranes. Now, I do not mean to say that such inflammation can always be prevented, or that the abstraction of very large quantities of blood will make the patient a better subject for it if it should occur; but it seems reasonable to suppose, and our experience of these cases, and of other cases bearing an analogy to them, confirms the opinion that there is less danger of inflammation where the antiphlogistic treatment has been carried to a moderate extent, and where the patient has been kept in a state of perfect quiet, than where bleeding and laxative medicines have been neglected, and the patient has been allowed to exercise his body and mind, and to live on his usual diet.

"The quantity of blood which the vessels of the brain contain depends very much on the position of the head with respect to the rest of the body. Not only in cases of concussion, but in all other cases where there has been an injury of the brain, or one likely to affect the brain, the head and shoulders should be raised by additional pillows, so that the blood may have an easy descent to the right side of the heart. In addition to this, in severe cases of concussion, the head should be shaved, and compresses should be applied, constantly moistened with a cold evaporating lotion. Opiates should be avoided. It is difficult to conceive what good purpose they can ever have been expected to answer; and at any rate they tend to constipate the bowels, and not unfrequently cause a confusion of symptoms, the patient complaining of headache, of which it is difficult to say whether it belongs to the injury itself or to the opium.

"In taking a view of the various satisfactory reasons which may be urged in favor of a particular plan of treatment in cases of concussion of the brain, we must not overlook the circumstance that this treatment may be carried too far; and we must endeavour to avoid the error which I have known some surgeons fall into, of resorting to a too free use of the lancet. At first, when the reaction of the heart has taken place, it may be right that the patient should lose a considerable quantity of blood, so as completely to subdue the force of the circulation. Afterwards, for the most part, it is only an occasional bloodletting that is required, and that to a moderate extent. It has appeared to me that this mode of proceeding has usually done more, both towards relieving the present symptoms and preventing subsequent inflammation, than a more active system of depletion; and where very large quantities of blood have been already taken away, if inflammation should shew itself, our resources are comparatively limited, and we are not able to meet it with that energy and vigor which the circumstances of the case require.

"Where bleeding has been carried to a great extent, symptoms frequently occur which in reality arise from the loss of blood; but which a superficial observer will be led to attribute to the injury itself, and concerning which, indeed, it is sometimes difficult even for the most experienced surgeon to pronounce, in the first instance, to which of these two causes they are to be referred. Repeated copious bloodletting is of itself adequate to produce a hardness of the pulse, which we shall in vain endeavour to subdue by persevering in the same system of treatment. In many individuals it will produce headache and confusion of mind, not very different from what the injury itself had previously occasioned. These things may be observed especially in young females who are disposed to hysteria, and whom I have often known to suffer from a continued aggravation of such symptoms as I have described, while the system of depletion has been continued; recovering immediately on the use of the lancet being laid aside, and on their being allowed to take solid nourishment, with occasional doses of the carbonate of ammonia."*

*Treatment to be employed in cases of compression of the brain, not complicated with wounds of the brain or its membranes.*—In all cases of injury of the head, if the dura mater is wounded, the danger is considerably aggravated. This circumstance also modifies, or even alters, the treatment. Mr. Brodie at present supposes that such a complication does not exist. When the symptoms of compression indicate danger, the cause on which they depend should be removed by a surgical operation, where it can be accomplished.

"An operation is also to be resorted to in those cases in which there are symptoms of pressure depending on hemorrhage between the dura mater and the bone. But here another question arises: What is the evidence which is to enable us to detect a mass of extravasated blood in this situation, and how

* Dr. Marshall Hall has published, in the thirteenth volume of the Medico-Chirurgical Transactions, some excellent practical observations on the effects of copious bloodletting, many of which are applicable to the cases mentioned above.

are we to determine what is the exact part of the cranium which should be perforated by the trephine? I must here refer to an observation which has been already made. Blood is seldom poured out in any considerable quantity between the dura mater and the bone, except in consequence of a laceration of the middle meningeal artery, or one of its principal branches; and it is very rare for this accident to occur except as a consequence of fracture. If, therefore, we find the patient lying in a state of stupor, and, on examining the head, we discover a fracture with or without depression, extending in the direction of the middle meningeal artery, although the existence of an extravasation on the surface of the dura mater is not thereby reduced to an absolute certainty, it is rendered highly probable, and the surgeon, under these circumstances, would neglect his duty if he omitted to apply the trephine. If it happens that no extravasation is discovered, the operation does not leave the patient in a worse condition than he was in before: but, if there be an extravasation, although it does not place him in a state of absolute security, it relieves the present symptoms, and gives him a chance of recovery which he would not have had otherwise.

"Where no fracture is discoverable, yet if there is other evidence of the injury having fallen on that part of the cranium in which the middle meningeal artery is situated, the use of the trephine may be resorted to on speculation, rather than that the patient should be left to die without an attempt being made for his preservation. I cannot, indeed, adduce any particular experience of my own in favor of what is here recommended; but I conceive that the instances which have been recorded, in which the middle meningeal artery has been ruptured without any fracture of the bone, and the known fact that there is sometimes a fracture of the inner table of the skull, while there is none of the outer table, sufficiently justify such an experiment in desperate cases, or even in those in which there is much danger. Our judgment may be assisted on those occasions by attending to the rule laid down by Mr. Abernethy: 'If there be so much blood on the dura mater as materially to derange the functions of the brain, the bone to a certain extent will no longer receive blood from within; and, by the operation performed for its exposure, the pericranium must have been separated from its outside. I believe that a bone so circumstanced will not be found to bleed; and I am certain that it cannot bleed with the same freedom and celerity as it does when the dura mater remains connected with it.'*

"In applying the trephine on account of a fracture with depression, the removal of a small portion of bone is generally sufficient; and there is, indeed, no sufficient reason for removing any considerable portion of the cranium. But, in resorting to the application of the trephine on account of an extravasation of blood on the surface of the dura mater, our practice should be different. The bone should be removed extensively, so as to expose at any rate a large portion of the surface of the dura mater, in which the extravasation has taken place. The necessity of attending to this rule was impressed on my mind by a case which came under my care in the hospital in the year 1814. A man was admitted with a fracture of the parietal bone, and a large extravasation of blood between the cranium and the dura mater. I removed two triangular pieces of bone with a straight saw, and a large quantity of blood, partly fluid, partly coagulated, escaped through the open-

* Abernethy on Injuries of the Head, edit. 1797, pp. 33, 34.

ing that was made. The symptoms under which the patient laboured were immediately relieved, and for several days he appeared to be going on favorably. But suppuration ultimately took place on the surface of the dura mater, wherever the extravasation had separated it from the bone. The opening made by the saw being in great measure occupied by granulations from the dura mater, afforded no opportunity for the free escape of the pus which was formed in the neighbourhood, in consequence of which the abscess burrowed between the dura mater and the bone, separating them from each other much farther than they had been separated originally. As soon as I had discovered what was taking place, I removed another portion of bone with the trephine; but the mischief had now become so extensive that the operation gave scarcely temporary relief, and the patient died. Reflecting on the case afterwards, I could not but acknowledge that if I had removed a larger portion of the bone in the first instance, so as to expose the extravasated blood more completely, the pus which was afterwards secreted could have been freely discharged, and the life of the patient would in all probability have been preserved.

"But the most common cause of pressure on the brain is an extravasation of blood within the cavity of the dura mater. Here, if there be any large collection of blood in one mass, it is generally in the basis of the cranium; sometimes in the substance of the brain, at other times in the cells between the tunica arachnoides and pia mater. In either of these cases it is beyond the reach of an operation. There may, indeed, be a large extravasation of blood on the superior surface of the cerebrum immediately beneath the dura mater; but, if such an extravasation does exist, in what manner are we to become informed of its existence? We may regard it as a general rule, that an operation is not applicable to cases of compression of the brain from internal extravasation. But there are few general rules in surgery to which some exceptions may not be made. Let us suppose a case in which a considerable portion of bone has already been removed; in which the dura mater is seen exposed, of a blue colour, lifted up by a collection of blood beneath it, and bulging as it were into the aperture which has been made in the cranium. Are we justified in puncturing the dura mater for the purpose of allowing the extravasation to escape? Every thing that we see of wounds of the dura mater tends to prove the very great danger of this kind of injury. The dura mater should never be wantonly punctured; but we cannot doubt that, in what may be regarded as desperate cases, it must be right to give the patient the chance, small as it may be, which the division of the dura mater affords him. The combination of circumstances which would lead to such an operation must be very rare; but it may occur nevertheless, and the surgeon should be prepared to meet it. The late Mr. Chevalier was called to a child a year and a half old, who had received a severe blow on the head. The child lay in a state of insensibility, and was affected with convulsions. There was no wound of the scalp, but, on an attentive examination of the head, the fontanel appeared to be somewhat elevated. Mr. Chevalier was led, therefore, to make a crucial incision of the scalp, by dissecting up the corners of which he exposed the fontanel. He then made an angular incision of the right side of the fontanel, and raised the membrane forming it so as to expose the surface of the dura mater, beneath which the purple colour of extravasated blood was plainly to be seen. A puncture being made carefully with a lancet, the blood issued at first with considerable force, spouting to the distance of a foot. Three or four ounces of blood escaped. The symptoms were immediately relieved, and the child recovered without any further unfavorable symptoms.*

"The following case, which is still more remarkable, was communicated to me by Mr. Ogle, of Great Russell-street, in whose practice it occurred some years ago:

"A woman, who kept a cellar in Monmouth-street for the sale of second-hand linen, &c. fell from the street, head foremost, to the bottom of the cellar. When taken up, she was in a state of total insensibility. Mr. Ogle, being immediately sent for, found her lying as if in a fit of apoplexy. He ordered her head to be shaved; and, on examining it afterwards, discovered no wound of the scalp, but observed that she flinched very much when pressure was made on one spot near the anterior and superior angle of one of the parietal bones. Having made an incision of the scalp at this part, he could perceive no appearance of fracture. Nevertheless, as the woman was manifestly in imminent danger, he thought it expedient to remove a portion of the bone with the trephine. Immediately on the bone being removed, the dura mater, of a dark colour, rose into the opening nearly as high as the external surface of the cranium. Convinced, from its appearance, and from the feeling of tension communicated to the fingers, that a fluid was interposed between it and the brain, and that that fluid was blood, Mr. Ogle ventured to puncture the dura mater with the point of a lancet. The puncture was instantly followed by a stream or jet of blood, which spirted out to the height of some feet. Immediately on the blood being discharged, the woman, who till that moment had continued totally insensible, opened her eyes. After looking about her, apparently amazed, she exclaimed, 'What is the matter? what are you doing with me?' and was able to give a clear account of the manner in which the accident had occurred. From this time she recovered without any untoward symptoms. It was impossible to ascertain the precise quantity of blood which escaped through the opening of the dura mater, but Mr. Ogle supposes it to have been about three-quarters of an ounce.

"But cases such as these are to be regarded as out of the common course of events. The ordinary cases of extravasation within the dura mater from injury are to be treated as we treat cases of apoplexy, or of paralytic seizure, in consequence of a blood vessel within the head being ruptured from disease; on the same principle as that on which we treat other cases of internal hemorrhage. Take blood from the arm so as to reduce the force of the heart's action. Repeat this, or take blood by cupping, as soon as the pulse has recovered from the effect of the former bloodletting; administer active saline purgatives; let the head be shaved and bathed with a cold lotion, being kept at the same time in an elevated position; and, although such a plan of treatment will not effect the cure of a patient who lies with stertorous breathing in a state of perfect stupor, many will recover under it in whom the symptoms of pressure have been very urgent. In some instances a slight improvement is perceptible from day to day, until, at the end of two or three weeks, the patient seems to be restored to his natural condition. In other instances his recovery is less complete, and a partial loss of nervous power may remain for many months; or such a memorial of the accident as a dilated pupil, a be-

* London Medical and Physical Journal, vol. viii. p. 505.

numbed hand, or a paralytic limb, may exist for a much longer period, for years, or even during the remainder of the patient's life."

There are many cases in which there is reason to believe that there is extravasation of blood within the cranium, although not in sufficient quantity to produce any formidable symptoms. It has been already observed that it is sometimes difficult to distinguish such cases from concussion of the brain. Fortunately, where the distinction is plain, it leads to no difference of treatment.

Fracture of the cranium frequently exists, with considerable depression of bone, while the patient suffers but slightly, or perhaps no symptoms at all exist. Here arises the important question, whether, under such circumstances, an operation should be performed for the purpose of removing the depression. From various facts which Mr. Brodie particularly enumerates, the following conclusion would be derived, that "it is most prudent to abstain from the use of the trephine where there is a fracture, with depression of the cranium, producing at the time no unfavorable symptoms." But much may be said on the other side of the question. Where a depression of the cranium is allowed to remain, it sometimes happens that symptoms arise after a considerable lapse of time, which may even endanger the life of the patient from the continuance of the depression, although it occasioned no inconvenience in the first instance. An instructive case in point is mentioned in which Sir Everard Home, after three years had elapsed from the time of the accident, was induced to remove nearly the whole of the depressed bone with the trephine. The symptoms which existed before the operation were immediately relieved. After a candid consideration of the question, Mr. Brodie states—

"Whatever may have been my first impression on the subject, it appears to me at this moment that the views of Sir Astley Cooper are well founded; and that in those cases in which a depression of bone exists without any symptoms, or with only trifling symptoms arising from it, the surgeon can follow no better general rule than this: if the depression be exposed in consequence of a wound of the scalp, let him apply the trephine, and elevate the depression; but if there is a depression without a wound of the scalp in consequence of the accident, let him not make such a wound by an operation. An exception may, perhaps, be properly made with respect to very extensive depressions of the cranium, which it may be prudent to expose and elevate at all events, not because there is a greater danger of suppuration from these than from smaller injuries, but on account of the ultimate ill consequences which the patient may experience if the brain be left permanently subjected to a very considerable pressure."

*Treatment of contusions and wounds of the scalp.*—Extravasation of blood in the cellular texture of the scalp seems to require, for the most part, no particular attention. Punctured and incised wounds of the scalp require, in the first instance at least, no peculiar treatment. Mr. Brodie knows no reason why the parts should not be brought together with adhesive plaster, as in wounds elsewhere. Erysipelas not unfrequently follows a wound of the scalp, but it seems to occur equally whether the wound is dressed with adhesive plaster or in any other manner. Sometimes the parts will unite by the first intention. In other cases there will be no adhesion; or the adhesions may be partial, one part of the wound uniting, with suppuration in another. In this case much attention is required, lest the formation of abscesses in certain places should do injury to the pericranium and bone, and destroy the adhesions in the neighbourhood.

*Treatment of fractures of the cranium unattended with depression* —It appears to be the general opinion of modern surgeons, that in these cases where there is no evidence of any considerable extravasation between the dura mater and the bone, nothing but a strict antiphlogistic regimen is required. The use of the trephine is here not necessary, notwithstanding the practice and opinions of Mr. POTT, whose doctrines upon this subject are canvassed and refuted by Mr. Brodie. Fractures of the cranium, however, even without compression, are always to be regarded with a jealous eye, especially where the scalp is wounded, and the pericranium separated from the bone. In these cases there is much danger of the formation of matter between the dura mater and the bone.

*Treatment of wounds of the brain and its membranes.*—" Although the condition of the patient who labours under a wound of the brain, or dura mater, is essentially different from that of one in whom no such wound exists, the general treatment required in these two orders of cases is nearly similar; and bleeding, purgatives, low diet, and a state of perfect repose, form an important part of the remedies to be employed in cases of wounds, as well as in those of concussion and compression of the brain.

" The object of the local treatment, where there is a wound of the brain or its membranes, is not so much to relieve the existing symptoms as to prevent future ill consequences, the principal of which are (as I shall shew hereafter), first, inflammation extending from the wound over the membranes of the brain, and producing an effusion of serum and pus; secondly, inflammation, suppuration, sloughing, and dissolution of the substance of the brain; thirdly, protrusion of the brain, in the form of what is commonly denominated a hernia cerebri.

" A judicious surgeon will always bear in mind that, especially on those occasions, the first rule of his art is not to add to the mischief already done. If splinters of bone have penetrated into the brain, and can be removed with perfect facility, and without the smallest additional disturbance to the injured organ, such removal cannot be improper, and may probably be useful. Many persons, however, have recovered, in whom an opposite practice has been pursued. I saw a gentleman in whom detached fragments of bone remained imbedded in the brain, many months after he had received a wound in the head from a pistol bullet, and who suffered scarcely at all from the injury. Do not such cases justify us in leaving splinters of bone untouched, where there is any kind of obstacle to their easy extraction? Are they not even sufficient to show that any other mode of proceeding would be improper, and that it is better to leave the patient to take his chance with the splinters lodged in the brain, than to commit the smallest additional violence in an endeavour to remove them?

" A similar observation may be made respecting depressions of bone when complicated with wound of the brain. If the edge of the depressed bone be imbedded in the substance of the brain, it may be proper to restore it to its natural level, provided that this can be readily accomplished with the forceps or elevator. But individuals have recovered, in whom a depression of bone has been allowed, under these circumstances, to remain without being elevated; and it cannot be advisable to risk this chance of recovery, whatever it may be, if the elevation requires the application of such a degree of force as is likely to cause the most trifling additional injury to the wounded brain. I have myself been led to doubt the expediency of applying the trephine in those cases in which there were no circumstances making the operation absolutely necessary. The motion of the saw must occasion more or less jar to the tender substance of the brain; and this, which may be of little consequence where the brain and its membranes are entire, may make a serious difference as to the degree of danger, where these parts are already lacerated and contused. There is, moreover, the same objection here as in other instances to the removal of any considerable portion of the parietes of the cranium, namely, the liability which it occasions to the formation of a hernia cerebri."

Mr. Brodie has not been able to discover, among all the works which he has consulted, a single instance of recovery from a wound of the posterior lobes of the cerebrum, of the cerebellum, or medulla oblongata; and in the great majority of cases in which a cure has taken place, the injury has been confined to the frontal bone, and that part of the brain which is covered and defended by it.

We do not remember to have perused any communication in which so great a mass of valuable practical information was condensed into so small a space. We earnestly recommend it to the attention of the student and practitioner.

